# Gaussian process regression model for normalization of LC-MS data using scan-level information

**DOI:** 10.1186/1477-5956-11-S1-S13

**Published:** 2013-11-07

**Authors:** Mohammad R Nezami Ranjbar, Yi Zhao, Mahlet G Tadesse, Yue Wang, Habtom W Ressom

**Affiliations:** 1 Department of Electrical and Computer Engineering, Virginia Tech, Arlington, VA, USA; 2 Lombardi Comprehensive Cancer Center, Georgetown University, Washington DC, USA; 3 Department of Biostatistics, Bioinformatics and Biomathematics, Georgetown University, Washington DC, USA; 4 Department of Mathematics and Statistics, Georgetown University, Washington DC, USA

**Keywords:** Extracted ion chromatogram (EIC), Evaluation, Gaussian process, Liquid chromatography-mass spectrometry (LC-MS), Normalization, Quality control (QC), Scan-level data

## Abstract

**Background:**

Differences in sample collection, biomolecule extraction, and instrument variability introduce bias to data generated by liquid chromatography coupled with mass spectrometry (LC-MS). Normalization is used to address these issues. In this paper, we introduce a new normalization method using the Gaussian process regression model (GPRM) that utilizes information from individual scans within an extracted ion chromatogram (EIC) of a peak. The proposed method is particularly applicable for normalization based on analysis order of LC-MS runs. Our method uses measurement variabilities estimated through LC-MS data acquired from quality control samples to correct for bias caused by instrument drift. Maximum likelihood approach is used to find the optimal parameters for the fitted GPRM. We review several normalization methods and compare their performance with GPRM.

**Results:**

To evaluate the performance of different normalization methods, we consider LC-MS data from a study where metabolomic approach is utilized to discover biomarkers for liver cancer. The LC-MS data were acquired by analysis of sera from liver cancer patients and cirrhotic controls. In addition, LC-MS runs from a quality control (QC) sample are included to assess the run to run variability and to evaluate the ability of various normalization method in reducing this undesired variability. Also, ANOVA models are applied to the normalized LC-MS data to identify ions with intensity measurements that are significantly different between cases and controls.

**Conclusions:**

One of the challenges in using label-free LC-MS for quantitation of biomolecules is systematic bias in measurements. Several normalization methods have been introduced to overcome this issue, but there is no universally applicable approach at the present time. Each data set should be carefully examined to determine the most appropriate normalization method. We review here several existing methods and introduce the GPRM for normalization of LC-MS data. Through our in-house data set, we show that the GPRM outperforms other normalization methods considered here, in terms of decreasing the variability of ion intensities among quality control runs.

## Background

Liquid chromatography coupled with mass spectrometry is one of the promising high through-put tools for identification and quantification of biomolecules extracted from serum, plasma, tissue, etc. Analysis of a sample by LC-MS typically generates three pieces of information: a pair of mass-to-charge ratio (*m/z*) and retention time (*RT*) along with a related ion intensity. Following preprocessing of data from a set LC-MS runs, a data matrix is created with each row and column representing a feature (*RT, m/z*) and a sample, respectively. Assuming, *pf eaturesandn *samples, we consider in this paper a *p *× *n *data matrix.

Normalization of the preprocessed LC-MS data is considered before statistical analysis to decrease undesired bias [1]. The bias can be from differences in sample collection, biomolecule extraction, or from column separation nonlinearity, ionization variability, etc [2]. The importance of the sample preparation step to achieve consistent results in different runs of the same experiment was emphasized in recent studies [3].

To the best of our knowledge, limited studies investigated the performance of existing normalization methods through real LC-MS data [2,4]. In these studies, a pooled mixture of multiple samples is utilized to generate replicate QC runs. Then, the QC runs are utilized to estimate and correct the bias.

In [5] we reviewed most of the existing methods for normalization of LC-MS data. Some of the methods were modified and all methods were employed to conduct an evaluation of their performances on a real data set. In this study, we expand the aforementioned work by introducing a new normalization method using Gaussian process regression to capture the variation of ion intensities. We use maximum likelihood approach to find the parameters for the fitted stochastic process. Then the learned model is used to correct for the drift based on analysis order. This approach can be used with either preprocessed data [6] or the raw data (scan-level data). The latter allows us to capture information that may be lost during preprocessing, but also deals with more complex data.

A data set generated from both experimental and QC samples is used here to assess normalization methods. We use the number of ions with significant variation within the QC runs as a measure to evaluate the run to run variability for each ion. From this point of view a normalization method is assumed to have better performance if it can decrease this variation for more ions. In addition, cross-validation is used to evaluate the performance of each normalization method. The methods are further compared based on their ability to detect statistically significant ions between cases and controls. In other words we look into different batches of data in the same experiment with dependent and independent set of samples and compare the normalization methods based on their ability to increase the number of statistically significant ions overlapping among different batches. However, we do not use this criteria to rank the methods as the ground-truth is not available. The variability with-in the QC runs is utilized to compare the GPRM with other normalization methods reviewed in this paper and particularly with those that use analysis order for normalization.

## Results and discussion

We propose a normalization method based on a Gaussian process regression model (GPRM). The method can be used for normalization of either integrated peak intensities or on the basis of scan-level intensities from EICs. We denote the former GPRM and the latter GPRM-EIC. The performance of GPRM and GPRM-EIC are compared with two different sets of normalization methods: analysis order-based normalization methods (Table 1) and other normalization methods reviewed in this paper (Table 2). Table 2 shows only the top three normalization methods with the highest performance.

**Table 1:** Performance comparison of analysis order-based normalization methods using QC runs and the number of statistically significant ions between cases and controls

Batch	Raw	LOESS	LOESS-CV	GPRM	GPRM-EIC	2D-GPRM-EIC
1 Positive	5.0	5.2	4.6	2.9	2.5	2.1
2 Positive	7.0	4.3	3.7	2.5	2.2	1.8

1 Negative	5.0	3.8	3.6	3.0	2.6	2.2
2 Negative	20	12	10.9	6.8	5.7	4.4

(A) Percentage of the number of ions with significant QC variation (*q*_ζ _< 0.1)						

**Mode**	**Raw**	**LOESS**	**LOESS-CV**	**GPRM**	**GPRM-EIC**	**2D-GPRM-EIC**

Positive	23	27	30	37	39	42

Negative	11	19	21	28	31	33

(B) Number of statistically significant ions between cases and controls, overlapping in batch B1 and B2						

**Table 2:** Performance comparison of TIC, MedScale, and Quantile normalization

Batch	Raw	TIC	MedScale	Quantile
1 Positive	5.0	6.1	5.5	4.0
2 Positive	7.0	4.1	3.5	3.0

1 Negative	5.0	3.1	4.1	2.9
2 Negative	20	11	9.9	7.3

(A) Percentage of the number of ions with significant QC variation (*q_ζ _*< 0.1)				

**Mode**	**Raw**	**TIC**	**MedScale**	**Quantile**

Positive	23	40	32	62

Negative	11	19	15	105

(B) Number of statistically significant ions between cases and controls, overlapping in batch B1 and B2				

By comparing all the reviewed methods across different batches in the data set, we observed that three methods, TIC, MedScale, and Quantile normalization, showed better performance consistently [5]. As shown in Tables 1 and 2, both GPRM and GPRM-EIC reduce the percentage of ions with *q_ζ _<*0.1 compared with other normalization method or unnormalized data (see Evaluation Method). This indicates that our proposed methods lead to a decrease in the number of features with significant variation across the QC runs.

**Figure 1:**
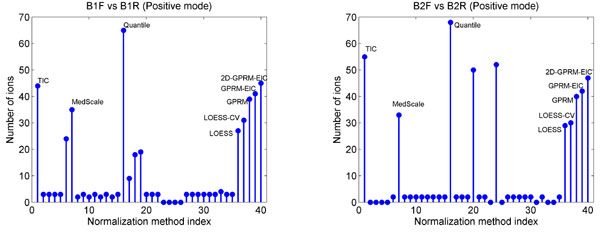
**Consistent methods**. Comparison of 39 evaluated normalization methods. We look for methods with consistent increase in the number of ions detected as statistically significant when comparing different batches of data. Each figure shows the number of selected ions based on two batches of data. In each figure the batches have the same set of samples.

Also we examined to find whether normalization helps with detection of new significant differences. This is done based on LC-MS data from multiple experiments with dependent and independent batches of sample sets (see LC-MS Dataset). By looking at within-batch combinations, i.e. dependent sets of samples, we can determine which method is consistent for the same set of samples in terms of finding more number of statistically significant features (Figure 1). On the other hand, considering between batch comparisons enables us to evaluate the methods based on independent samples (Tables 1 and 2). In general for the same method with different modifications, the decrease in variance of *ζ *in equation (20), leads to more number of ions selected as statistically significant.

From Table1t can be seen that among analysis order-based normalization methods, our proposed approach has the highest efficiency in decreasing the variability within the QC runs. Table 1 shows that these methods also outperformed other normalization methods by considering the same measure for estimated variability of QC runs. We think that GPRM can perform better as it handles the drift by using a stochastic model and optimization to find the parameters. In comparison, other analysis order-based normalization methods work with limited possible values for parameters and as a result they may not reach the highest possible performance. Also by taking advantage of the scan-level intensities from EICs, GPRM-EIC is able to achieve better performance than GPRM. However, GPRM-EIC requires appropriate alignment of the scan-level peaks. Finally, by merging information across different scans, 2D-GPRM-EIC showed the best performance.

Comparing Tables 1 and 2 reveals that although some methods show less decrease within QC variability, but they lead to more number of ions selected as statistically significant between cases and controls. For example Quantile method reduced the percentage of the ions with *q_ζ _<*0.1 to 4.0% and 3.0% for B1 and B2 respectively in positive mode (Section: Evaluation Methods). In comparison GPRM achieved 2.5% and 2.2%. However the number of ions selected as statistically significant between cases and controls are 62 and 37 for Quantile and GPRM respectively in positive mode. As pointed out before, the ground truth is not available to evaluate the performance of the normalization methods on the basis of detected differences between cases and controls. Thus, all ions found statistically significant are regarded as potential candidates until subsequent verification is conducted to determine if the differences are true or biologically meaningful. However, the LC-MS runs are expected to yield better reproducibility following normalization. In particular, the QC runs in this study are anticipated to have the least variability, at least for a considerable subset of the analytes.

## Conclusions

Systematic bias is one of the challenges in quantitative comparison of biomolecules by LC-MS. Various normalization methods have been proposed to address this issue. However, there no universally applicable solutions at the present time. Thus, each LC-MS data set should be carefully inspected to determine the most appropriate normalization procedure. Since most of the evaluation studies have been performed on data from relative a small sample size without adequate replicates and QC runs, additional investigations on large-scale LC-MS data are needed [5].

We reviewed several existing normalization methods in this paper. Also a new method for normalization of LC-MS data is introduced. The method uses the analysis order information in a Gaussian process model. Compared to other methods that also use analysis order information, our model has some advantages. It can model the bias from instrument drift more efficiently as a statistical approach is used which includes noise in the model to estimate the parameters through optimization. Therefore it is more precise in estimation of the scale parameter compared to some analysis order-based methods which search heuristically for the span parameter of the smoothing algorithm. In addition we extended this method to perform normalization on the basis of EICs obtained from raw LC-MS data instead of the preprocessed peak list.

We evaluated the performance of the GPRM and other existing normalization methods using our in-house LC-MS data generated from both experimental (cases and controls) and QC samples. The QC runs were used to estimate and correct the drift in the ion intensities. The normalization methods were assessed based on two criteria: (1) the decrease in the within-sample variability; (2) the number of extra ions selected as statistically significant compared to those obtained without normalization. The first criterion is used to rank the models based on their performance, while the second criterion used to investigate the effect of normalization in terms of the number of possible candidates with significant differences between cases and controls. Our method showed improvement over existing methods considering the first criteria. However some methods with a lower rank, e.g. Quantile, provide more number of candidates. Therefore it is required to conduct a verification experiment to confirm the true differences and discard false positives.

While the performance of the normalization method can be improved by using the scan-level LC-MS data following appropriate alignment, there are some issues. One of these issues is misalignment of the peaks across the scans. We used a simple approach to align the peaks, but more advanced techniques are available to further improve the alignment. Also, including prior distributions on the parameters of the GPRM through Bayesian analysis can potentially elevate the performance of our method. Future work will focus on addressing these issues.

### Methods

Several normalization techniques have been proposed for LC-MS data. As normalization is a well-known concept in the area of genomics, most of the methods have been adapted from the techniques developed for gene expression microarray data [4,7-10]. Usually the underlying assumption of these approaches is that the average biomolecule concentrations should be equal for all samples in the same experiment. To examine the performance of these methods, replicate LC-MS runs of a reference sample can be used [5].

In this paper, we introduce a Gaussian process regression model for normalization based on analysis order. Also we extend this method to estimate variability of scan-level ion intensities within an EIC of a peak. For comparison we investigated the following normalization methods: (i) normalization based on total ion count (TIC), (ii) median scale normalization, (iii) pretreatment methods such as scaling, centering and transformation, (iv) normalization based on internal standards, (v) quantile normalization, (vi) MA transform linear/local regression normalization, (vii) normalization based on QC consistency, (viii) normalization based on stable features, and (ix) normalization based on analysis order. We implemented these methods and in some cases we modified the algorithm [5].

As described in Background, the data matrix X_p × n _is made of n samples and p features, where each sample is represented as a column vector **x***_j _*for *j *= 1,..., *n*, so **x***_j _*= [*x*_1*j *_.. *x_pj_*]*^T ^*and:

$${\text{X}}_{p\times n}=\left[{\text{x}}_{1}\;..\;{\text{x}}_{n}\right]$$

### Existing normalization methods

*Normalization based on TIC *is the most common and the simplest approach, which divides all ion intensities of a LC-MS run by the area under the TIC curve, where TIC is the total energy or the sum of all intensities of related *m/z *values for each *RT *(scan):

(1) $$TIC_{j}\left(rt\right)\;=\;\overset{}{\underset{\forall \;m/z}{\sum }}I_{j}\left(rt,\;m/z\right)$$

and for the normalization factor:

(2) $$E_{j}\;=\;\overset{}{\underset{\forall \;rt}{\sum }}TIC_{j}\left(rt\right)$$

where *I_j _*is the intensity of the pair (*rt, m/z*) for the *j*th sample. Here both TIC of the sample and TIC of the selected ions can be used. We preferred the latter as the former includes all the noisy ions which have been already removed in the preprocessing step.

*Median scale normalization *[7] considers one LC-MS run as a reference, then all other LC-MS runs are scaled based on the median of the ratio of all intensities to the reference:

(3) $${\tilde{\text{x}}}_{j}\;=\;\frac{{\text{x}}_{j}}{\underset{i}{\text{median}}\;\left(\frac{x_{ij}}{x_{i}^{*}}\right)}$$

where *x_i_*^∗ ^is the *i*th ion of the reference sample **x**^∗^. Similarly *x_ij _*is the *i*th ion of the *j*th sample **x_j_**. We modified this method by adopting some rules to select the reference. The first option is to select one of the QC runs. In the second scenario, any sample may be selected as the reference. In both cases it is convenient to choose randomly, but we decided to include the option to select the reference sample based on minimum number of missing values/outliers, where the outliers are detected based on projection statistics.

*Pretreatment methods*, including three classes (class I, II, III) have been proposed for normalization of LC-MS data [11]. The choice of a pretreatment method depends on the properties of the data set and the biological problem to be explored. Class I is centring by subtracting the average of each ion from all intensities of that ion to remove the offset. This is not enough when the data is from different distributions. Class II consists of five different scaling approaches (auto, range, Pareto, vast, and level scaling). Autoscaling is applied after centering by normalizing data with a scale measure *s_i_*, such as standard deviation. Additionally range scaling is defined as:

(4) $${\tilde{x}}_{ij\;}=\;\frac{x_{ij}-{\bar{x}}_{i}}{\text{max}\left(x_{ij}\right)-\;\text{min}\left(x_{ij}\right)}$$

Pareto scaling tries to reduce the relative importance of large values but keep the data structure partially intact and calculated as ${\tilde{x}}_{ij}\;=\;\left(x_{ij}-{\bar{x}}_{i}\right)/\sqrt{s_{i}}$. The disadvantage of this method is its high sensitivity to outliers. If we are interested in ions with small fluctuations we may use vast scaling as:

(5) $${\tilde{x}}_{ij}\;=\;\frac{1}{CV_{i}}\frac{x_{ij}-{\bar{x}}_{i}}{s_{i}}$$

where the coefficient of variation (CV) is defined as $CV_{i}\;=\;s_{i}/{\bar{x}}_{i}$. Level scaling focuses on relative responses and suited for identification of biomarkers:

(6) $${\tilde{x}}_{ij}\;=\;\frac{x_{ij}-\;{\bar{x}}_{i}}{{\bar{x}}_{i}}$$

The drawback of this method is inflation of measurement errors. In class III, usually centered log magnitude or square root of intensities is used to reduce the effect of different data distributions and make skewed distributions more symmetric, but this class has difficulties with zero values and large variances.

*Normalization based on internal standards *is another popular approach for LC-MS data [12]. In this method by inserting one or more internal standards with controlled amounts of concentration, normalization is done based on the variation of these landmarks. If there is only one standard available, one sample is considered as the reference, then we scale all samples by the ratio of the standard's intensity of the reference to the standard's intensities of the samples. This approach can be modified by selecting a robust value for the reference to avoid accepting outliers as standard ions. If there are more than one internal standard, two approaches are feasible. First by using a distance measure we can find the closest standard to each ion and apply the previous method with one standard. Moreover it is possible to find a regression model for the variation of standards versus order of ions and apply the result to all intensities. The problem with this method is that it is expensive because it needs to add internal standards with precise concentrations in the sample preparation phase. This approach performs well when the correlation between an ion and the internal standard is not high, otherwise it does not meaningful to be used for normalization.

*Quantile normalization *is one of the nonparametric techniques which was first introduced for normalization of Affymetrix gene expression arrays [10]. In [4,13], this method has been applied to proteomic data. The aim of this method is to make the distribution of intensities the same across all samples. It can be implemented in three steps by creating a mapping between ranks and values:

(1) Find the smallest values for each vector or array. Save the average or median of these values.

(2) Similarly, find the second smallest values, and up to the n smallest values for each vector or array. Save the averages or medians of these values.

(3) For each vector or array, replace the sorted actual values with these averages, and resort them again.

**Figure 2:**
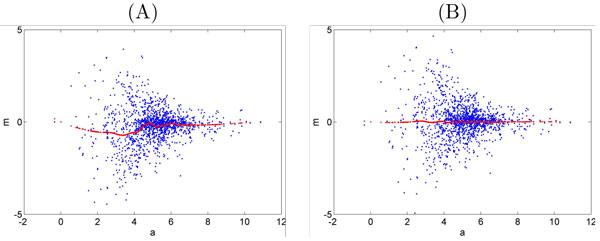
**MA transform**. MA-transform plot before (A) and after (B) normalization, one sample vs the reference sample. The curves in the figures represent $\hat{m}$ values used in equation (9) estimated by regression.

*MA transform linear regression normalization *assumes that systematic bias is linearly dependent on ion intensities. First, MA-transformed scatter plots are constructed as in Figure 2 with *M *and *A *defined as in [10]:

(7) $${m_{i}}^{\left(j,k\right)}=\text{ log }x_{ij}-\text{log }x_{ik}$$

and

(8) $${a_{i}}^{\left(j,k\right)}\;=\;\frac{1}{2}\left(\text{log}\;x_{ij}\;+\;\text{log}\;x_{ik}\right)$$

where *j ≠ k *and *j, k ∈ *{1*, .., n*}. Then two choices are possible to proceed with normalization: (i) to consider one sample or the mean/median of all samples as the reference; (ii) to run the algorithm pairwise for all the samples until convergence, i.e. when the change of overall maximum intensity of the data set is less than a certain threshold in (7) and (8). It is common to define **x***_k _*as the average (or median for the robust estimation) of all samples. Then the algorithm normalizes the log magnitude of ion intensities by subtracting the peak ratios calculated by applying least squares regression to M versus A plots:

(9) $${{\tilde{m}}_{i}}^{\left(j,k\right)}\;=\;{m_{i}}^{\left(j,k\right)}\;-\;{{\hat{m}}_{i}}^{\left(j,k\right)}$$

where $\hat{m}$ is the estimated vector from the regression model. The main idea of this method is to enforce the average log intensity difference of all peaks to zero.

*MA transform local regression normalization *is MA transform linear regression, but instead of using a linear model, it applies piecewise linear or other nonlinear models such as higher order polynomials and splines to find the baseline curve of intensities. Also locally weighted polynomial regression (LOESS) technique [14] has been used to smooth the log magnitude of relative ion intensities versus the reference or in a pairwise manner (Figure 2).

**Figure 3:**
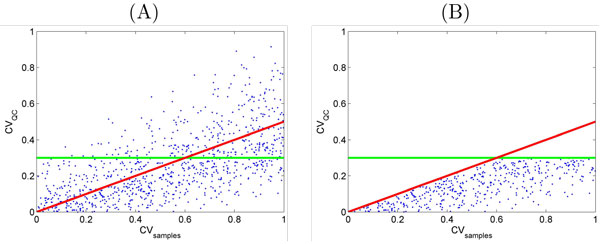
**CV screening**. Coefficient of variation of ions for QC samples versus others. Feature cleaning based on criteria (C1) and (C2). ions located above either of the two lines (A) are removed as they do not show consistency (B).

*Normalization based on QC consistency *uses a different rationale for normalization of LC-MS data [15]. If a data set involves QC runs of a sample (e.g. a reference sample or a mixture of pooled samples) and the QC runs are included in between different analyses, it is reasonable to expect less variation in ion intensities for QCs compared to other samples. Then the consistency of each ion is evaluated based on two criteria. Feature cleaning accept an ion if it meet both criteria, otherwise it is rejected and will not be used in next processing levels including normalization:

$$\begin{array}{c} \left(\text{C1}\right)\;CV_{i}^{QC}<TH_{1}\\ \left(\text{C2}\right)\;CV_{i}^{S}>TH_{2}\times CV_{i}^{QC} \end{array}$$

where $CV_{i}^{QC}$ and $CV_{i}^{S}$ are CV of *i*th ion for QCs and all samples except QCs respectively (Figure 3). However it is not clear how to select *TH*_1 _and *TH*_2 _and they should be determined for each data set separately.

*Normalization based on stable features *[16] tries to find a subset of stable ions and then normalize based on the variation of those ions in each sample. Assuming *T*_CV _as the maximum acceptable threshold, stable features are found as:

$${x_{ij}}^{SF}\;=\;\left\{x_{ij}:\;CV_{i}\leq T_{CV}\right\}$$

These features are used for normalization as follows:

(10) $${\tilde{\text{x}}}_{j}\;=\;\frac{{\text{x}}_{j}}{\underset{i}{\mathrm{median}}\;\left({\text{x}}_{ij}^{SF}\right)}$$

*Normalization based on analysis order *is one of the most recent approaches [2]. The main idea of this method is to model the variation of intensities versus the sample's run (injection) order in the experiment, and to remove this variation by applying a smoothing regression technique. This approach needs a set of reference samples to model the variation versus analysis order based on their deviation from expected intensity values. However this method has not been examined with a large data set and only a set of technical replicates was used in the work reported in [2]. Also only animal samples with few numbers of known proteins were used in the study. The authors reported that their normalization method outperformed all other existing methods in their experiment.

**Figure 4:**
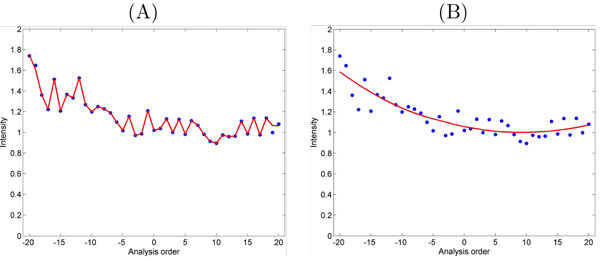
**Smoothing parameter**. Effect of different smoothing parameters: small value close to 0 (A), large value close to 1 (B).

**Figure 5:**
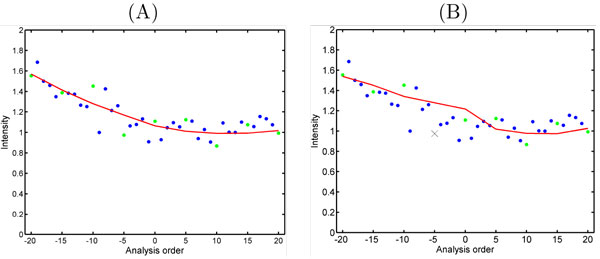
**LOO with LOESS**. LOESS method applied to the intensities of QCs versus analysis for one ion (A). An example of leave-one-out cross-validation to find a proper choice of span parameter (B).

This method can be expanded to include all samples in an experiment by applying the smoothing algorithm without any reference ions or samples. However, it is difficult to decide how much smoothing is enough for a particular ion. For example, it is not clear how the proper choice of span parameter can be determined for the LOESS algorithm (Figure 4). The authors proposed to use a certain range of span parameters in [0, 1] and try to find the best value by visual inspection or by using quantitative measures such as coefficient of variation (CV). In [15] cross validation methods such as leave-one-out (LOO) are recommended to select the best value for span parameter (Figure 5). It is suggested to consider a discrete subset of all possible values in [0, 1], then to perform cross validation to find the best value only on this subset which is not the optimal approach.

### Proposed normalization method

One way to address the issue of selecting the smoothing parameter of the LOESS algorithm, is to use a stochastic model and use optimization to learn the parameters. We propose a stochastic model to correct for drift using the analysis order information in which the LC-MS data were generated. This method uses preprocessed data to perform normalization [6]. Next, we extend the method to apply normalization using scan-level data.

#### Normalization based on Gaussian process (GPRM)

Considering preprocessed data, we assume ion intensities across different runs are observations from a Gaussian random process:

(11) $$x^{\left(i\right)}\left(t\right)\sim GP\left(\mu^{\left(i\right)}\left(t\right),{\text{Σ}}^{\left(i\right)}\left(t,t^{\prime }\right)\right)$$

for *t ∈ *{*t*_1 _*.. t_n_*} which is the analysis order index. In addition index *i *points to the *i*th ion. To use the model in (11), all we need is to find the co-variance matrix of the process **Σ**^(*i*)^.

For a given ion with intensities **x**(**t**), we consider a parametric model for estimation of covariance matrix by using a kernel function *k *as:

(12) $${\text{Σ}}_{j_{1}j_{2}}=\text{ cov }\left(x\left({t_{j}}_{{}_{\text{1}}}\right),\;x\left({t_{j}}_{{}_{\text{2}}}\right)\right)\;=k\left({t_{j}}_{{}_{\text{1}}},\;{t_{j}}_{{}_{\text{2}}}\right)$$

**Figure 6:**
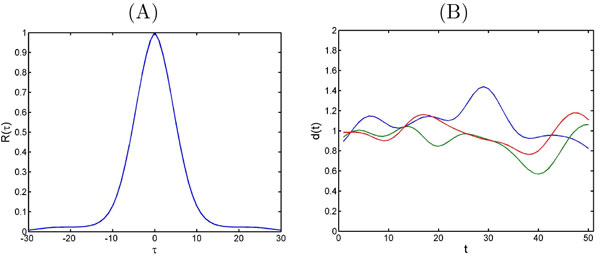
**Gaussian process**. An example of a kernel function based on equation (13) (A). Three examples of ensemble functions generated using this kernel (B).

It is reasonable to consider that the drift is highly correlated for adjacent runs, as it is caused by instrument variations in ion count measurements. Also drifts in ion intensities should be less similar for distant runs. As the covariance matrix is positive definite, the kernel function should have certain properties to generate a valid covariance matrix as an estimate of the real covariance matrix. In [17] Gaussian process $\left(GP\right)$ is used for regression and a series of valid kernel functions are introduced. We selected the Matern kernel which enables us to control the desired smoothness properties by changing its parameter *v*:

(13) $$R\left(\tau \right)=\frac{2^{1-\upsilon }}{\text{Γ}\left(\upsilon \right)}{\left(\frac{\sqrt{2\upsilon \tau }}{\ell }\right)}^{\upsilon }K_{\upsilon }\left(\frac{\sqrt{2\upsilon \tau }}{\ell }\right)$$

where $\tau =\;|t_{j_{1}}-t_{j_{2}}|,\;\Gamma \left(\right)$ is the Gamma function, and K*_v_*() is the modified Bessel function (Figure 6) and we have $k\left({t_{j}}_{{}_{\text{1}}};{t_{j}}_{{}_{\text{2}}}\right)=\sigma^{\text{2}}R\left(\tau \right)$. Here ℓ  is the scale parameter which determines the degree of correlation between runs based on their distance in terms of analysis order and usually *υ *∈ {1*/*2, 3*/*2, 5*/*2}. To consider noise in our regression model, we include an additional term to the kernel function:

(14) $${\text{Σ}}_{j_{1}j_{2}}=\sigma^{2}R\left(\left|t_{j_{1}}-t_{j_{2}}\right|\right)+\sigma_{\in }^{2}\delta \left(t_{j_{1}}-t_{j_{2}}\right)$$

where $\sigma_{\in }^{2}$ is the variance of the zero mean Gaussian noise.

For a given ion, we assumed intensities as observations of a GP, thus they follow a multivariate normal distribution, i.e. if $\text{x}={\left[x\left(t_{1}\right)\;..\;x\left(t_{n}\right)\right]}^{T}$then $\text{x}\;\sim \;N\left(\mu ,\sum \right)$. It is reasonable to consider $\mu \left(t\right)=\mu_{0}+\mu_{1}t$ as a linear function of time which explains the linear component of the drift while the nonlinear part is modelled by ∑. Therefore for each ion we have:

(15) $$P\left(\text{x}|\theta ;\text{t}\right)=\frac{\text{exp}\left(-\frac{1}{2}{\left(\text{x}-\mu_{\theta }\right)}^{T}\sum_{\theta }^{-1}\left(\text{x}-\mu_{\theta }\right)\right)}{\sqrt{{\left(2\pi \right)}^{n}|{\text{Σ}}_{\theta }|}}$$

where $\theta ={\left[\ell \sigma \;\sigma_{\varepsilon }\;\mu_{0}\;\mu_{\text{1}}\right]}^{T}$ is the vector model parameters. We can estimate the parameters of the kernel from the covariance matrix by using maximum likelihood method. To maximize the likelihood function of *θ*, we minimize  L as:

(16) $$L\left(\theta \right)=-\text{log}P\left(x|\theta ;t\right)$$

Any optimization method can be used to find the maximum likelihood estimator, *θ^∗^*. For example, using gradient descent approach: $\theta \left(r+\text{1}\right)=\theta \left(r\right)-\lambda \nabla_{\theta }L$, where *r *is the iteration index and 0 *< λ <*1.

Once $\theta_{QC}^{*}$ is derived for $t_{QC}=\left\{t_{1},..,t_{n_{QC}}\right\}$ for *n_QC _*quality control samples, we can estimate drift for *t *= 1, .., *n*. The estimated regression curve which models the variability of the QCs is used to correct the drift for other samples similar to what is performed by using the results from LOESS algorithm. A concern here is the risk of overfitting due to the presence of several parameters in the model while using only the QC runs as the reference. To address this concern, we use regularization. The regularization term is defined as a function of the scale parameter in our kernel function to reflect the consistency for measurements from both QC runs and the ones from experimental samples. Therefore, first we find the scale parameter for experimental samples by using the same approach. Then regularization is performed by applying a constraint based on scale parameters, i.e.$\ell_{QC}>\ell_{S}$, and Karush-Kuhn-Tucker (KKT) approach is used to find $\theta_{QC}^{*}$:

(17) $$L\left(\theta_{QC},n\right)=-\text{log}P\left(\text{x}|\theta_{QC};t_{QC}\right)+\eta \left(\ell_{S}-\ell_{QC}\right)$$

So far, we explained the algorithm for a given ion. As we have multiple ions, we repeat the pro-cedure for each ion separately to estimate $\theta^{\left(i\right)}={\left[\ell^{\left(i\right)}\sigma^{\left(i\right)}\;\sigma_{\in }^{\left(i\right)}\;\mu_{0}^{\left(i\right)}\;\mu_{1}^{\left(i\right)}\right]}^{T}\text{for }x^{\left(i\right)}\sim N\left(\mu^{\left(i\right)},{\text{Σ}}^{\left(i\right)}\right)$

#### Gaussian Process Regression Model - Extracted Ion Chromatogram(GPRM-EIC)

**Figure 7:**
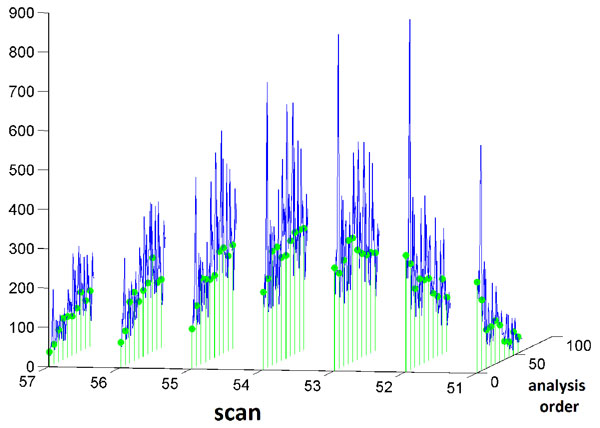
**Drift in individual scans 1**. Several scans of the same ion from different samples. The solid line shows the variation for experimental samples while the solid dots represents the peak intensities for the QC runs.

**Figure 8:**
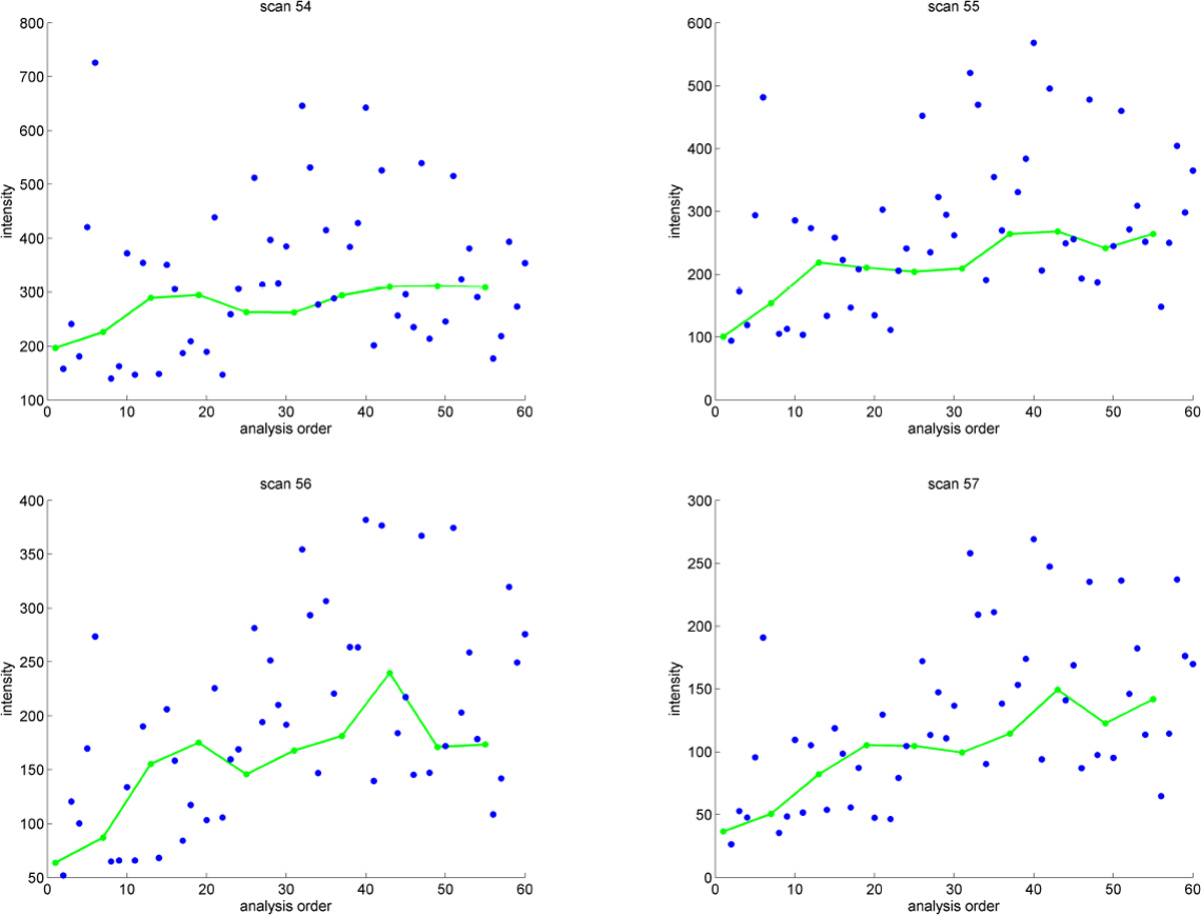
**Drift in individual scans 2**. Four scans from a single EIC. The solid line shows the variation of the QC runs while the dots represents the peak intensities for experimental samples.

All the methods introduced so far, use single peak intensity derived from each EIC (typically by calculating the area under EIC curve). However GPRM-EIC takes advantage of the scan level ion intensities within an EIC (Figure 7). Specifically, GPRM-EIC looks into individual scans to monitor the variations based on analysis order (Figure 8). This can model the changes observed in the intensities of scan-level of QC samples and based on that correct for experimental samples' ion intensities. To apply the correction we used the integral of the differences of ion intensities before and after correction.

Therefore we employ the model in (11) and update that to include scan-level information:

(18) $$x^{\left(i,s\right)}\left(t\right)\sim GP\left(\mu^{\left(i,s\right)}\left(t\right),{\text{Σ}}^{\left(i,s\right)}\left(t,\;t^{\prime }\right)\right)$$

for *t *= *t*_1_*, .., t_n_*, where index *i *points to the *i*th ion and *s *represents the scan number. To use the model in (18), similar to the previous model, all we need is to find the covariance matrix of the process **Σ**. Here ℓ  and *µ *parameters are defined as scalars.

To summarize, first we find the base peaks for the corresponding mass of each ion to form the EIC. This can be done by using XCMS2, to find regions of interest [18] (ROI) for the ions selected in pre-processing. However we can use segmentation along mass axis as it is employed in original XCMS [19]. Thereafter, by looking into raw data, each individual scan is used to model the drift based on analysis order. The model is used to correct for the variation. Finally the normalized peak intensities are used to recalculate the area under the EIC curve and update the ion intensities. One issue here is the misalignment of the peaks. The drift in each scan may be partly due to retention time differences across samples. To correct for this we simply align the first peak in each spectrum to match the scans across different samples.

#### 2-D Gaussian Process Regression Model - Extracted Ion Chromatogram (2D-GPRM-EIC)

In the method above, we analyzed each scan separately. It is possible to consider a 2 dimensional GP to combine the information from different scans for each peak from all QCs:

(19) $$x^{\left(i\right)}\left(z\right)\sim GP\left(\mu^{\left(i\right)}\left(z\right),{\text{Σ}}^{\left(i\right)}\left(z,z^{\prime }\right)\right)$$

where **z **= [*t s*]*^T ^*for analysis order $t=t_{1},..,t_{n_{QC}}$ and scan $s=s_{1}^{\left(i\right)},\;..,\;s_{S\left(i\right)}^{\left(i\right)}$. Here *S*(*i*) is the number of scans for ion *i*. By using this model, we consider two different scales along analysis order and scan time axes and use a 2-D Gaussian process to model the variability.

### LC-MS data

We used in-house LC-MS data set to evaluate the normalization methods described in the previous sections. The data set is derived from three types of samples, cases, controls, and QCs. The samples were collected from adult patients at Tanta University Hospital, Tanta, Egypt. The participants consist of 40 hepatocellular carcinoma (HCC) cases and 50 patients with liver cirrhosis. Through peripheral venepuncture single blood sample was drawn into 10 mL BD Vacutainer sterile vacuum tubes without the presence of anticoagulant. The blood was immediately centrifuged at 1000 *× *g for 10 min at room temperature. The serum supernatant was carefully collected and centrifuged at 2500 *× *g for 10 min at room temperature. After aliquoting, serum was kept frozen at -80 °C until use.

**Figure 9:**
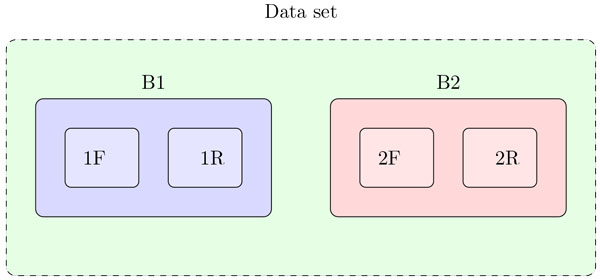
**Data set**. Structure of the data set.

The samples were run in two different batches, B1 and B2, and in each batch two sets of experiments were included, the "forward" (F) and the "reverse" (R) experiments (Figure 9). The forward order experiment includes all the samples in B1, and the reverse order experiment includes the same samples as in forward experiment, but the run order is reversed. Each batch includes 10 QC samples, 20 cases and 25 controls. For each experiment, QCs were made of pooling case samples from the same batch. QCs were injected every other 5th run. For cases and controls the injection is done alternating between samples from each group to reduce bias [20].

The data were acquired using ultra performance liquid chromatography (UPLC) coupled with QTOF MS in both positive and negative modes. The raw data were preprocessed by XCMS package [19]. The details of the data set can be found in [21].

### Evaluation criteria

The main assumption of any normalization method is reproducibility of the experiment. Here we assume that at least a considerably large subset of the measured values is reproducible. A measured value refers to a single peak which represents an ion intensity across different runs. Since the QC runs are expected to be identical, their measurements can be used to estimate instrument variability and to assess the reproducibility of the experiment.

Coefficient of variation (CV) is commonly used as a measure of performance, which is defined as the ratio of standard deviation to absolute mean for the *i*th ion: *CV_i _*= σ_i_/|µ_i_|. As there are more than one ion to evaluate, the median *CV *for each group of samples, or the number of ions with *CV *less than a certain threshold, is typically used to evaluate reproducibility. Based on this definition, we expect a decrease in *CV *at least for QC runs when applying any normalization method. However using *CV *to evaluate normalization methods is tricky, as smaller *CV *does not necessarily imply better normalization due to the presence of differentially abundant ions. Thus a method that ensures the efficiency in finding true biological differences is desired. In this study, we evaluate the impact of the normalization method in terms of decrease in within-QC variation in each batch of data. In addition, we use the number of statistically significant ions between cases and controls as a metric to compare normalization methods. We consider this measure only when the increase in number of detected ions is consistent between batches with dependent and independent sets of samples. As we have duplicate experiments of the same phenotypes, we evaluated the performance of the normalization methods by cross validation between different experiments. A one-way repeated measures ANOVA model is used to investigate the performance of different methods based on the variation of QCs across different experiments:

(20) $$x_{ijk}^{QC}=\mu_{i}+\alpha_{ij}+\zeta_{ik}+\in_{ijk}$$

where $x_{ijk}^{QC}$ is the intensity of *k*th QC run for *i*th ion in batch *j *and *ζ *is the random effect so that $\forall i:E_{k}\left[\zeta_{ik}\right]=0.$

A normalization method is evaluated on the basis of the number of ions with reduced variance of *ζ_ik _*. We evaluate this by using the *F *test for the ratio of the sum of squares from *ζ *to the sum of the squares of ∈ which is the unexplained variation or error. To correct for the multiple testing effect, we use *q_ζ _<*0.1, where *q *is FDR-adjusted p-value estimated using the Storey method [22].

Furthermore the number of statistically significant ions in each data set is compared for each dataset before and after normalization. A two-way repeated measures ANOVA is used to analyze the combined forward and reverse experiments (e.g. Exp 1F & Exp 1R):

(21) $$x_{ijkl}=\mu_{i}+\alpha_{ij}+\beta_{ik}+\gamma_{ijk}+\xi_{ijl}+\in_{ijkl}$$

for ion *i *in sample *l *from group *j *in batch *k*. The group effect *α*, and the batch effect *β*, are considered in the model as well as the possible group-batch interactions *γ *while *ξ *models the within sample variation. Also a two-way ANOVA is used to analyze the between-batch combinations (e.g. Exp 1F & Exp 2F):

(22) $$x_{ijkl}=\mu_{i}+\alpha_{ij}+\beta_{ik}+\gamma_{ijk}+\in_{ijkl}$$

ions with significant group-batch interaction, i.e. *q*_γ _*<*0.1, were removed from the analysis, where *q *is FDR-adjusted p-value estimated using the Storey method [22]. Significant ions are selected based on *q_i,α _≤ *0.1.

## Competing interests

The authors declare that they have no competing interests.

## Authors' contributions

MRNR designed the study, analyzed and interpreted the data, participated in the statistical analysis and drafted the manuscript. YZ participated in the statistical analysis. YW and MGT drafted the manuscript. HWR guided the study and drafted the manuscript. All authors read and approved the final manuscript.

## Declarations

The publication costs for this article were funded by the corresponding author.

This article has been published as part of *Proteome Science *Volume 11 Supplement 1, 2013: Selected articles from the IEEE International Conference on Bioinformatics and Biomedicine 2012: Proteome Science. The full contents of the supplement are available online at http://www.proteomesci.com/supplements/11/S1.
